# Mouse Astrocytes Promote Microglial Ramification by Releasing TGF-β and Forming Glial Fibers

**DOI:** 10.3389/fncel.2020.00195

**Published:** 2020-07-10

**Authors:** Jinqiang Zhang, Lijuan Zhang, Saini Yi, Xue Jiang, Yan Qiao, Yue Zhang, Chenghong Xiao, Tao Zhou

**Affiliations:** ^1^Resource Institute for Chinese & Ethnic Materia Medica, Guizhou University of Traditional Chinese Medicine, Guiyang, China; ^2^School of Life Science and Technology, Center for Informational Biology, University of Electronic Science and Technology of China, Chengdu, China; ^3^Institute of Medical Biology Science, Chinese Academy of Medical Science, Beijing, China

**Keywords:** astrocyte, microglia, glial fibrillary acidic protein, microglial ramification, TGF-β

## Abstract

The morphology of microglial cells is often closely related to their functions. The mechanisms that regulate microglial ramification are not well understood. Here we reveal the biological mechanisms by which astrocytes regulate microglial ramification. Morphological variation in mouse microglial cultures was measured in terms of cell area as well as branch number and length. Effects on microglial ramification were analyzed after microinjecting the toxin L-alpha-aminoadipic acid (L-AAA) in the mouse cortex or hippocampus to ablate astrocytes, and after culturing microglia on their own in an astrocyte-conditioned medium (ACM) or together with astrocytes in coculture. TGF-β expression was determined by Western blotting, immunohistochemistry, and ELISA. The TGF-β signaling pathway was blocked by the TGF-β antibody to assess the role of TGF-β on microglial ramification. The results showed that microglia had more and longer branches and smaller cell bodies in brain areas where astrocytes were abundant. In the mouse cortex and hippocampus, ablation of astrocytes by L-AAA decreased number and length of microglial branches and increased the size of cell bodies. Similar results were obtained with isolated microglia in culture. However, isolated microglia were able to maintain their multibranched structure for a long time when cultured on astrocyte monolayers. Ameboid microglia isolated from P0 to P3 mice showed increased ramification when cultured in ACM or on astrocyte monolayers. Microglia cultured on astrocyte monolayers showed more complex branching structures than those cultured in ACM. Blocking astrocyte-derived TGF-β decreased microglial ramification. Astrocytes induced the formation of protuberances on branches of microglia by forming glial fibers that increased traction. These experiments in mice suggest that astrocytes promote microglial ramification by forming glial fibers to create traction and by secreting soluble factors into the surroundings. For example, astrocyte-secreted TGF-β promotes microglia to generate primitive branches, whose ramification is refined by glial fibers.

## Introduction

Microglia are derived from primitive myeloid progenitors in the yolk sac and maintain a self-renewing population throughout the life of the organism (Singhal and Baune, 2017; Perry, 2018). In addition to secreting inflammatory mediators, microglia play important roles in inhibiting apoptosis, removing myelin, or Aβ, as well as in promoting synaptic pruning, neurogenesis, and changes in neuronal circuitry and network connectivity (Perry, 2018). These functions of microglia depend on cytoskeletal rearrangement. The cytoskeletal rearrangements of microglia lead to morphological changes that allow phagocytosis, migration, and synaptic pruning (Hellwig et al., 2016; Shibata and Suzuki, 2017). In the healthy adult brain, microglia have a small soma with many fine branches, and the branches of “resting” microglia continuously survey the surrounding environment and interact with neurons and astrocytes (Santos et al., 2017; Facci et al., 2018). As microglia age, their ramified dendritic arbor shrinks significantly, with fewer branches and shorter total branch lengths (Ma and Wong, 2016; Niraula et al., 2017). These morphological changes in microglia are related to the pathology of neurodegenerative and psychiatric diseases, including Alzheimer’s disease and depression (Nayak et al., 2014; Benatti et al., 2016; Plescher et al., 2018).

Primary microglia have been used as an *in vitro* model of neuroinflammation for nearly 20 years. Clues to factors that affect microglial ramification have emerged from studies of differences between their morphology in primary culture and their morphology in the brain. In primary culture, most microglial cells exhibit ameboid morphology without branches, while some have several simple branches (Giulian and Baker, 1986). In contrast, microglia in the brain have a more complex branching structure, characterized by multiple branches protruding from small somata (Yuan et al., 2017; Zhang et al., 2017). This difference is due to the complexity of the brain’s internal environment, which includes several interacting cell populations and soluble factors (Silverman and Wong, 2018).

For example, astrocytes play an important role in regulating microglial ramification and function (Kalla et al., 2003; Schilling et al., 2001). Culturing ameboid microglia on astrocyte monolayers or by themselves in astrocyte-conditioned medium (ACM) causes their ramification (Schilling et al., 2001). ACM also upregulates proteins with antioxidant and anti-inflammatory activities in primary microglia, such as IL-10 (Madry et al., 2018) and TGF-β (Norden et al., 2014). However, a detailed understanding of how astrocytes regulate microglial ramification is lacking, in part because of the difficulty in using primary cultures to clarify events in the complex environment of the brain.

Therefore, the aim of this study was to investigate the role of astrocytes on microglial ramification by microinjecting the astrocytic toxin L-alpha-aminoadipic acid (L-AAA) into the cortex and hippocampus to ablate astrocytes, then examining microglial ramification. In addition, microglia were cultured by themselves in ACM or cocultured with astrocytes, and their ramification was compared. We provide evidence that astrocytes regulate microglial morphology through contact-dependent and -independent pathways. TGF-β from astrocytes plays a primary role in remodeling microglial ramification, and refinement of microglial ramification depends on direct contact with astrocytes. These data provide new insights into glial cell function.

## Materials and Methods

### Animals

C57BL/6J mice (*n* = 33, 29 males, four females) that were 8 weeks old and weighed 18–22 g were obtained from the Laboratory Animal Center of Sichuan Academy of Medical Sciences (Chengdu, China). All mice were housed under standard conditions (12-h light/12-h dark cycle, 22–26°C) with free access to food and water. Five male animals were used for morphological analysis of microglia in different brain regions. Ten male animals were used for ablation of astrocytes in the hippocampus, including five control and five experimental animals. Ten male animals were used for ablation of astrocytes in the cortex, including five control and five experimental animals. Four males and four females were used to breed newborn mice for cell culture. Cells obtained from the same litter were used for statistical analysis.

All animal experiments were approved by the Ethics Committee of the Guizhou University of Traditional Chinese Medicine and carried out in strict accordance with the US National Institutes of Health Guide for the Care and Use of Laboratory Animals (8th edition, revised 2010, National Research Council, Washington DC, USA).

### Astrocyte Ablation Using L-AAA

L-AAA (0.7 mg/ml; Sigma-Aldrich, St. Louis, MO, USA) was dissolved in 100% ethanol and stored at −20°C. Stock solutions of L-AAA were diluted with pyrogen-free physiological saline as vehicle (Otsuka Pharmaceutical, Tokushima, Japan). Twenty-four mice were equally and randomly divided into two groups of control mice and L-AAA mice, using a randomization table. A stainless steel cannula (25-gauge) was implanted into the hippocampus in mice anesthetized with 5% pentobarbital sodium (0.05 mg/kg, i.m., Germany; Research Resource Identifier, RRID:WS_20100520) using a stereotaxic apparatus. Freely moving mice received bilateral cortex or hippocampus injection of L-AAA (100 μg/μl, once daily for 2 days, 0.1 μl/min for 6 min; Lee et al., 2013). Sham mice injected with physiological saline were used as controls.

### Behavioral Testing

#### Novel Object Recognition Test

Mice were individually placed for 5 min in a Plexiglas arena (40 × 60 cm, walls 30 cm high), and exploration was quantified by video tracking software (OFT100). Subsequently, mice were subjected to three habituation sessions, in which two objects identical in shape, color, and odor were introduced into the arena for 3 min with a 2-min intertrial interval. Before the last session, one of the objects was replaced with a novel object. Time spent in exploration of each object was scored during each session.

#### Tail Suspension Test

Each mouse was individually suspended by 1 cm from the tip of the tail with adhesive tape from a ledge 30 cm above the floor of the cage. The whole process was recorded with a high-definition camera for 6 min. An observer masked to treatment conditions recorded the latency between suspension and the first abandonment of the struggle and immobility times for 6 min.

#### Forced Swim Test

At 24 h before the test, each mouse was individually placed in a glass cylinder (height: 25 cm, diameter: 15 cm) filled with 26°C water to a depth of 15 cm for 15 min. The next day, the mice were placed once again in the same situation for 6 min. The whole process was recorded with a high-definition camera. An observer was blinded to the test, and the immobility time was recorded during the last 4 min.

#### Open Field Test

Mice were placed into the open field (50 × 50 cm) and allowed to explore freely for 15 min. Total distance and time spent in the center (25 × 25 cm) were quantified using video tracking software (OFT100, Taimeng Tech Ltd., Chengdu, China).

### Isolation of Microglia and Astrocytes From Adult Mouse Brain

Cerebral hemispheres were isolated and homogenized to single-cell suspensions. Microglia were isolated on a Percoll density gradient as described (Grabert and Mccoll, 2018). Isolated microglia were seeded on astrocyte monolayers. Morphological changes in microglia were detected by immunohistochemistry (see “Immunohistochemistry” section).

### Cell Culture

#### Primary Microglial Culture

Primary microglial cultures were prepared as described (Zhao et al., 2017). In brief, cerebral hemispheres were obtained from postnatal (P0–P3) C57BL/6 mice. Brain tissues were cut into pieces and digested into single-cell suspension by 0.25% pancreatin (Gibco, Waltham, MA, USA; RRID:25200-056). Cell suspensions were filtered using a 70-μm cell strainer. The obtained mixed glial cells were cultured in DMEM/F12 (Gibco; RRID:C11330500BT) containing 10% fetal bovine serum (Gibco; RRID:A3160802) at 37°C in an atmosphere of 5% CO_2_. Microglia were mechanically isolated from the mixed glial cultures by shaking on a rotary shaker at 260 rpm for 4 h at 37°C. Floating microglia were collected and transferred to a culture dish. The purity of primary microglia was more than 98% detected by immunohistochemistry.

#### Microglial Culture in ACM

Primary astrocytes were isolated from P0–P3 mice as described (Lian et al., 2016). The purity of primary astrocyte was more than 95% detected by immunohistochemistry. Enriched astrocytes were plated in 24-well dishes at a density of 1 × 10^5^ cells/cm^2^, then cultured for 48 h. ACM was collected and centrifuged at 1,200 *g* for 5 min. Enriched microglia (1 × 10^5^ cells/cm^2^) were maintained in ACM at 37°C in an atmosphere of 5% CO_2_ for 24 h. Morphological changes of microglia were detected by immunohistochemistry (see “Immunohistochemistry” section).

#### Microglial Culture in Neuron-Conditioned Medium

Neurons were cultured as described (Zhang et al., 2017). Enriched neurons were placed in 24-well plates and cultured for 7 days. A neuron-conditioned medium was collected and centrifuged at 1,200 *g* for 5 min. Enriched microglia (1 × 10^5^ cells /cm^2^) were maintained in the neuron-conditioned medium at 37°C in an atmosphere of 5% CO_2_ for 24 h. Morphological changes of microglia were detected by immunohistochemistry (see “Immunohistochemistry” section).

#### Transwell Microglial Culture Experiments With Astrocytes or Neurons

Astrocytes or neurons were plated in the upper chamber of transwell plates (Corning Life Sciences, RRID:FK-cn014) at a density of 1 × 10^5^ cells/cm^2^. Primary microglia were plated in the lower chamber of the transwell plate at the same density. Plates were incubated at 37°C in an atmosphere of 5% CO_2_ for 24 h. Morphological changes of microglia were detected by immunohistochemistry (see “Immunohistochemistry” section).

#### Microglia–Astrocytes or Neuron Coculture Experiment

Astrocytes or neurons were plated in the 24-well plate at 1 × 10^5^ cells. After 7 days, primary microglia were plated in the upper of astrocytes or neurons at 1 × 10^5^ cells. These cells were incubated at 37°C in a 5% CO_2_ incubator for 24 h. Morphological changes of microglia were detected by immunohistochemistry (see “Immunohistochemistry” section).

#### Anti-TGF-β Treatment

Anti-TGF-β antibody (0.2 μg/mL, Arigo, City, China, ARG56078) or control IgG as negative control was added to primary microglial cultures. These cells were incubated at 37°C in an atmosphere of 5% CO_2_ for 24 h. Morphological changes of microglia were detected by immunohistochemistry (see “Immunohistochemistry” section).

### Preparation of Mouse Brain Slices

Mice were anesthetized with 10% pentobarbital and transcardially perfused with 0.9% saline containing heparin. Brains were removed, fixed in 4% paraformaldehyde for 48 h, washed with saline, and cryoprotected in 30% sucrose as described (Zhang et al., 2017). Sagittal sections containing the hippocampus and prefrontal cortex (20 μm thick) were obtained using a sliding vibratome (CM1900; Leica Microsystems, Wetzlar, Germany). Six sequential slices were placed in each well of a 12-well plate containing phosphate-buffered saline (PBS) with 0.02% sodium azide and stored at 4°C. One section containing either hippocampal or cortical tissue was selected from each well (i.e., one of every six sequential slices) as described (Zhang et al., 2017) and processed for immunohistochemistry (see “Immunohistochemistry” section).

### Cell Fixation

Cultured cells were placed on poly-lysine-coated slides for morphological analysis. These cells were washed three times using PBS and fixed with 4% paraformaldehyde for 30 min. Cells were then washed three more times with PBS.

### Immunohistochemistry

Brain slices or cultured cells were permeabilized with 0.5% Triton X-100 in PBS for 15 min, blocked in 10% donkey serum for 2 h, and incubated with primary antibodies (GFAP; 1:500; Cell Signaling Technology, Danvers, MA, USA; RRID:GA5), goat antibody against Iba-1 (1:400; Abcam, Cambridge, UK; RRID:ab5076), rabbit antibody against MAP2 (1:400; GeneTex, Irvine, CA, USA; RRID:GTX133109), and rabbit antibody against TGF-β 1 (1:400; Abcam, Cambridge, UK, RRID:ab92486). Cells were then incubated with DyLight 488-conjugated donkey anti-mouse secondary antibody (1:400, Jackson ImmunoResearch, West Grove, PA, USA; RRID:715-545-150), DyLight 594-conjugated donkey anti-goat antibody (1:400, Jackson ImmunoResearch, West Grove, PA, USA; RRID:705-585-147), or DyLight 488-conjugated donkey anti-rabbit antibody (1:400, Jackson ImmunoResearch, West Grove, PA, USA; RRID:711-545-152). Cells were then stained for 5 min with DAPI (1:10, 000; Roche, Basel, Switzerland; RRID:10236276001) to label nuclei.

### Analysis of Microglial Morphology

The morphology of microglia that had been prepared as mentioned in “Immunohistochemistry” section was analyzed using ImageJ 1.45 (National Institutes of Health, Bethesda, MD, USA) from micrographs at 20–40× magnification. In brief, microglial images were adjusted to observe all microglial processes *via* standard background subtraction (50 pixels with the sliding parabola option), and single-pixel background fluorescence was eliminated. Then, resulting images were converted to a binary image and skeletonized. The “Analyze Skeleton” plugin was used to analyze all skeletonized images, and soma area, number of somata per field, number of branches, and maximum branch length were determined. At least nine random fields on at least three separate coverslips were analyzed for each treatment.

### RNA Isolation and Gene Expression Analysis

RNA was isolated from the primary microglia, hippocampus, or cortex using the TRIzol (Invitrogen Life Technologies, Waltham, MA, USA) and chloroform extraction method, then purified with the Qiagen RNeasy kit (Takara, Japan). cDNA reverse transcription was performed using a high-capacity cDNA conversion kit (Takara, Japan). Quantitative RT-PCR (Bio-Rad CFX96, USA) was performed, and the threshold amplification cycle number (Ct) was determined for each reaction in the linear phase of the amplification plot. Each sample was tested in triplicate. Changes in gene expression were determined by the –ΔΔCt method. The values were normalized to β-actin. Primer sequences were as follows: β-actin, forward: 5′-CCGTGAAAAGATGACCCAGATC-3′, reverse: 5′-CACAGCCTGGATGGCTACGT-3′; TNF-α, forward: 5′-TACTGAACTTCGGGGTGATTGGTCC-3′, reverse: 5′-CAGCCTTGTCCCTTGAAGAGAACC-3′; IL-6, forward: 5′-ACCGCTATGAAGTTCCTCTC-3′, reverse: 5′-CTCTGTGAAGTCTCCTCTCC-3′; and IL-10, forward: 5′-TGGCCCAGAAATCAAGGAGC-3′; reverse: 5′-CAGCAGACTCAATACACACT-3′. Data were reported as fold increase in mRNA levels in treated samples relative to control.

### Enzyme-Linked Immunosorbent Assay (ELISA)

Levels of TNF-α, IL-6, TGF-β, and IL-10 in tissue lysate were quantified using mouse ELISA kits (QuantiCyto, Wuhan, China), according to the manufacturer’s protocols. Absorbance was measured at 450 nm using a microplate reader. Values were calculated as picograms per milliliter.

### Western Blotting and Densitometric Analysis

Proteins were extracted from PFC and hippocampus in lysis buffer (Solarbio, Beijing, China) for 20 min on ice, followed by centrifugation at 14,000 *g*. Total protein concentration was determined by BCA assay (Bosterbio, Wuhan, China). Total protein (100 μg) was separated on a 12.5% SDS-PAGE gel, then transferred to a nitrocellulose membrane. Membranes were blocked for 1 h in TBS-T (10 mM Tris, 150 mM NaCl, 0.05% Tween-20), followed by incubation overnight at 4°C with primary antibodies against TGF-β 1 (1:600; Abcam, UK, RRID:ab92486), pSMAD 2, 3 (1:100, Santa Cruz Biotech, CA, USA), SMAD 3 (1:100, Abcam, MA, USA), and β-actin (A5060, Sigma, MO, USA) antibodies diluted in 5% skim milk in 1× PBST and incubated for overnight at 4°C. HRP-conjugated secondary antibodies (1:5,000, Santa Cruz Biotech, CA, USA) were incubated for 1 h at room temperature and developed using the ECL Plus Western Blotting detection system (GE Healthcare) and imaged on a Bio-Rad ChemiDoc XRS.

### Statistical Analysis

Data were expressed as mean ± SEM from at least three independent experiments, with each treatment in duplicate or triplicate. Statistical analyses were performed using SPSS 17.0 (IBM, Chicago, USA). Differences between two means were evaluated using the paired Student’s *t*-test for independent samples, while differences among more than two means were evaluated using one- or two-way analysis of variance (ANOVA) followed by a *post hoc* Tukey’s multiple-comparison test. Differences were considered significant when *P* < 0.05.

## Results

### GFAP^+^ Cells Are Involved in Microglial Ramification in the Brain

**Figure 1:**
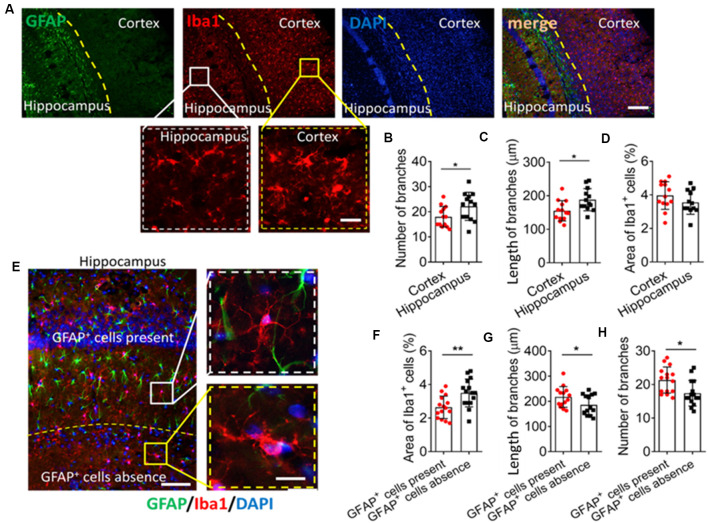
GFAP^+^ cells are involved in microglial ramification in the brain.** (A)** Fluorescence micrographs showing microglia and astrocyte in the mouse cortex and hippocampus. Microglia were labeled by Iba1 (red), astrocytes were labeled by GFAP (green), and nuclei were labeled by DAPI (blue). Scale bar, 100 μm. The zoomed insets highlight differences in microglial morphology between the cortex and hippocampus. Scale bar, 20 μm. (**B–D)** The number **(B)** and length **(C)** of branches, and percentage of Iba^+^ cells **(D)** in the hippocampus and cortex.** (E)** Fluorescence micrographs showing microglia and astrocytes in mouse hippocampus. Microglia were labeled by Iba1 (red), astrocytes were labeled by GFAP (green), and nuclei were labeled by DAPI (blue). Scale bar, 50 μm. The zoomed insets highlight differences in microglial morphology in regions where GFAP^+^ cells were present or absent. Scale bar, 10 μm.** (F–H)** Percentage of Iba^+^ cells **(F)**; length **(G)** and number **(H)** and of branches in regions where GFAP^+^ cells were present or absent (*n* = 4–5 animals/group). Results of each group were obtained from 4 independent samples, and 5–6 micrographs were collected from each sample. All Iba1^+^ cells in each micrograph were measured. Each dot in the bar graph represents the average of all Iba1^+^ cells in a micrograph. Data are presented as mean ± SEM (*n* = 20–24), *P < 0.05 and **P < 0.01 vs. corresponding controls (unpaired *t*-test).

We first examined differences in morphology between cultured microglia and microglia within the hippocampus. Microglia in primary culture were ameboid, but the hippocampal microglia had multiple branches (Supplementary Figure S1). We then tried to investigate differences in the local environment of microglial cells by immunohistochemistry. We found that Iba1^+^ cells had more and longer branches in the hippocampus. GFAP^+^ cells were more abundant in the hippocampus than in the cortex (Figures 1A–C). There was no significant difference in morphology or number of Iba1^+^ cells between the hippocampus and the cortex (Figure 1D).

It may be too difficult to clarify the role of GFAP^+^ cells on microglial ramification if two regions as functionally different as the hippocampus and cortex are compared. Therefore, we compared two regions within the hippocampus showing a significant difference in the number of GFAP^+^ cells. The area around the granule cell layer (GCL) of CA1 showed more GFAP^+^ cells than where the CA1 and dentate gyrus (DG) meet (Figure 1E), and Iba1^+^ cells in the area around the GCL had more and longer branches as well as smaller cell bodies (Figures 1F–H).

### Chemical Ablation of Astrocytes Induced an Ameboid Phenotype in Microglia in the Hippocampus and Cortex

**Figure 2:**
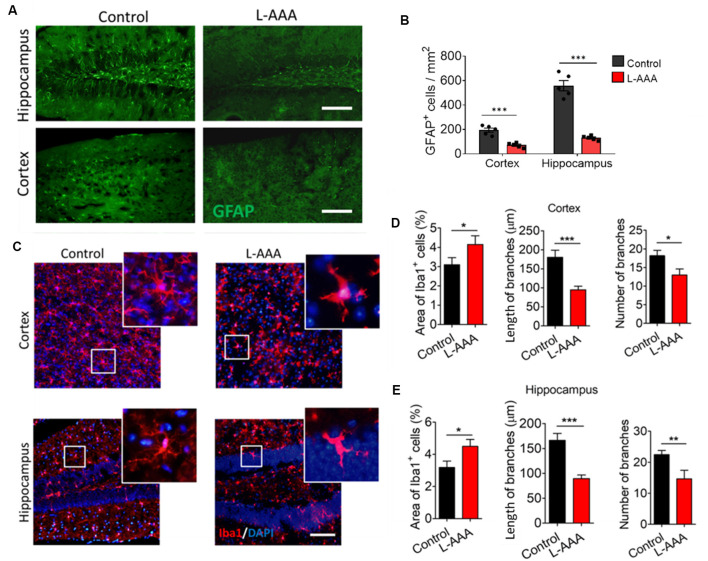
Ablation of astrocytes results in ameboid microglia in mouse hippocampus and cortex. **(A)** Fluorescence micrographs showing astrocytes in cortex and hippocampus from L-alpha-aminoadipic acid (L-AAA)-treated or control mice. Astrocytes were labeled by GFAP (green). Scale bar, 100 μm. **(B)** Quantification of GFAP^+^ cells in the hippocampus and cortex. **(C)** Fluorescence micrographs showing microglia in the cortex and hippocampus from L-AAA-treated or control mice. Microglia were labeled by Iba1 (red) and nuclei by DAPI (blue). The zoomed insets highlight differences in microglial morphology in the hippocampus of L-AAA-treated mice or control mice. Scale bar, 50 μm. **(D)** Percentage of Iba1^+^ cells, and length and number of branches in the cortex from L-AAA-treated or control mice. **(E)** Percentage of Iba1^+^ cells, and length and number (J) of branches in the hippocampus from L-AAA-treated or control mice. Data are presented as mean ± SEM (*n* = 12). *P < 0.05, **P < 0.01 and ***P < 0.005 (unpaired *t*-test).

Animals received L-AAA stereotactic injection in the hippocampus and cortex to induce astrocyte ablation through inhibition of glutamate synthesis. L-AAA treatment significantly reduced the number of GFAP^+^ cells in both hippocampus and cortex (Figures 2A,B). Immunofluorescence staining showed that ablation of astrocytes reduced the number and length of Iba1^+^ cell branches and induced an ameboid phenotype in microglia of both hippocampus and cortex (Figures 2C–E).

### Ablation of Astrocytes Results in Depressive-Like Behaviors, Cognitive Impairs, and Neuroinflammation

**Figure 3:**
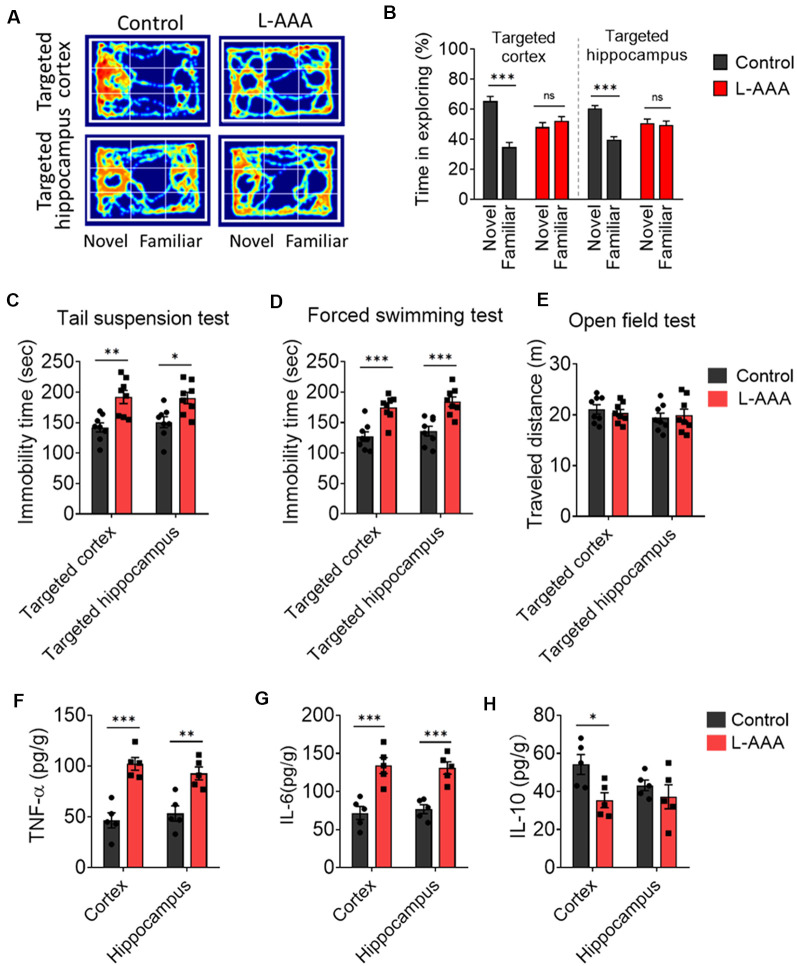
Ablation of astrocytes results in depressive-like behaviors, cognitive impairs, and neuroinflammation.** (A,B)** The novel object recognition test was used to evaluate cognitive function. Results in panels **(B)** reflect the results of 8–9 mice per group. Data are mean ± SEM. ***p < 0.005; unpaired *t*-test. **(C–E)** Depressive-like behaviors were measured in the **(C)** tail suspension test, **(D)** forced swimming test, **(E)** open-field test. Results in panels **(C–E)** reflect the results of eight mice per group. **(F–H)** Protein expression of inflammatory cytokines (TNF-α, IL-6, and IL-10) in the cortex and hippocampus. Results in panels **(F–H)** reflect the results of five mice per group. Data are mean ± SEM. *p < 0.05, **p < 0.01, ***p < 0.005; two-way ANOVA. ns: not significant (*p* > 0.05).

We observed the effect of astrocytes ablation on behavior and cognition through a battery of tests. Cognition was tested through the novel object recognition test (Figures 3A,B). The mice received ablation of astrocytes in the cortex or hippocampus, which exhibited cognitive defects compared to control mice (Figure 3B). We verified behavioral despair by performing a forced swimming test and tail suspension test. The results showed that ablation of astrocytes increased the immobility time of mice in the forced swimming test and tail suspension test (Figures 3C,D) but did not affect the distance travelled when compared to the control group (Figure 3E).

The protein levels of several key pro-inflammatory markers, including TNF-α and IL-6, were significantly elevated in the cortex and hippocampus of L-AAA-injected mice. The protein levels of the anti-inflammatory marker (IL-10) was significantly decreased in the cortex of L-AAA-injected mice (Figures 3F–H).

### Astrocytes Maintain Multibranched Structures in Microglia Isolated From the Brain

**Figure 4:**
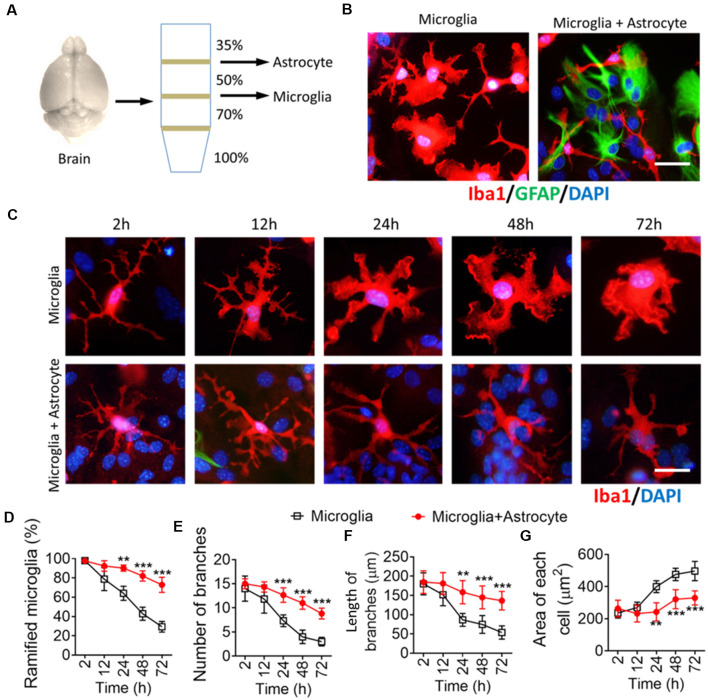
Astrocytes maintain a multibranched structure of microglia isolated from the brain. **(A)** Schematic diagram showing a Percoll density gradient for the isolation of microglia and astrocytes from the mouse brain. **(B)** Representative immunofluorescence micrographs showing morphological differences in microglia isolated from the brain cultured alone or cocultured on an astrocyte monolayer. Microglia were labeled by Iba1 (red), astrocytes were labeled by GFAP (green), and nuclei were labeled by DAPI (blue). Scale bar, 10 μm. **(C)** Time series of fluorescence micrographs showing morphological changes in microglia isolated from the brain after culture alone or coculture on an astrocyte monolayer. Microglia were labeled by Iba1 (red) and nuclei by DAPI (blue). Scale bar, 10 μm. **(D–F)** Time course of percentage of ramified microglia **(D)**, number **(E)**, and length **(F)** of branches, and cell body area of microglia **(G)**. Results of each group were obtained from four independent samples, and 5–6 micrographs were collected from each sample. All Iba1^+^ cells in each micrograph were measured. Data are presented as mean ± SEM (*n* = 4). **P < 0.01 and ***P < 0.005 vs. microglia (two-way ANOVA with LSD).

To confirm the contribution of astrocytes to microglial ramification, isolated microglia from adult mice were cultured alone or seeded onto astrocyte monolayers (Figures 4A,B). When isolated microglia were cultured alone, their branches had retracted into lamellar pseudopodia by 72 h, developing an ameboid morphology (Figures 4C,D). In contrast, when isolated microglia were cultured on astrocyte monolayers, their multibranched structure was maintained throughout 72 h, and they showed a higher percentage of ramified microglia, more and longer branches, and smaller somata than microglia cultured alone.

### Astrocytes Regulate Microglial Ramification Through Contact-Dependent and -Independent Mechanisms

**Figure 5:**
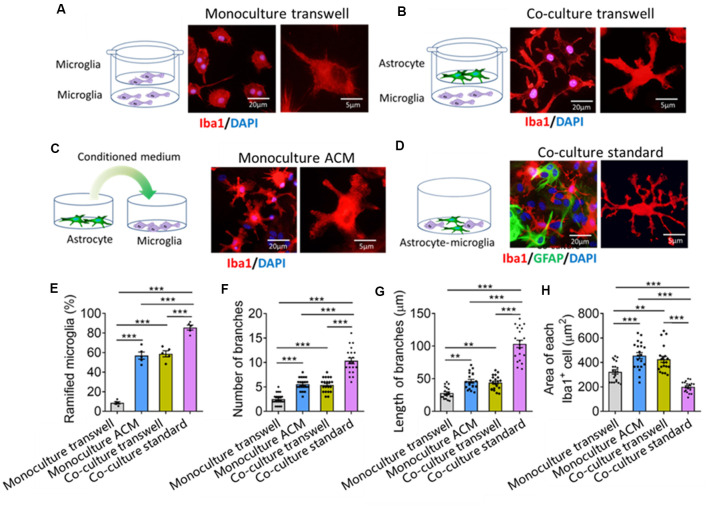
Astrocytes regulate microglial ramification through contact-dependent and -independent mechanisms. **(A–D)** Schematic diagrams showing four methods of culturing primary microglia: cultured alone **(A)**, cocultured with astrocyte by transwell **(B)**, cultured by astrocyte conditioned medium (ACM, **C**), and mix cultured with astrocyte **(D)**. Fluorescence micrographs showing different morphologies of microglia under different conditions. Microglia were labeled by Iba1 (red), astrocytes by GFAP (green), and nuclei by DAPI (blue). **(E–G)** Percentage of ramified microglia **(E)**, number **(F)** and length **(G)** of branches, and cell body area of microglia **(H)**. Results of each group were obtained from four independent samples, and 5–6 micrographs were collected from each sample. All Iba1^+^ cells in each micrograph were measured. Each dot in the bar graph represents the average of all Iba1^+^ cells in a micrograph. Data are presented as mean ± SEM (*n* = 20–24). **P < 0.01 and ***P < 0.005 between groups (one-way ANOVA with LSD).

To find out how astrocytes regulate microglial ramification, we prepared four types of primary microglial cultures: microglia alone in fresh medium in transwell dishes, microglia alone in ACM in standard culture dishes, cocultures of microglia with astrocytes in transwell dishes, and cocultures in standard culture dishes. Microglia cultured alone were mostly ameboid (Figure 5A), but microglia cultured alone in ACM or together with astrocytes in transwell dishes developed several primary branches (Figures 5B,C). Microglia cultured on astrocyte monolayers formed many delicate branches (Figure 5D).

Quantitative analysis showed that the percentage of ramified microglia and the number and length of branches were significantly higher in microglia cocultured with astrocytes or cultured alone in ACM than in microglia cultured alone in fresh medium (Figures 5E–H). Microglia cultured on astrocyte monolayers showed the highest percentage of ramified microglia, the most and longest branches, and the smallest cell bodies.

We also examined whether neurons also contributed to the remodeling of microglial branches. We found no significant differences in the percentage of ramified microglia, number of branches, or soma area between microglia cultured alone in fresh or neuron-conditioned medium or microglia cocultured with neurons in transwell dishes (Supplementary Figure S2).

### Astrocytes Secrete TGF-β That Promotes Microglial Ramification

**Figure 6:**
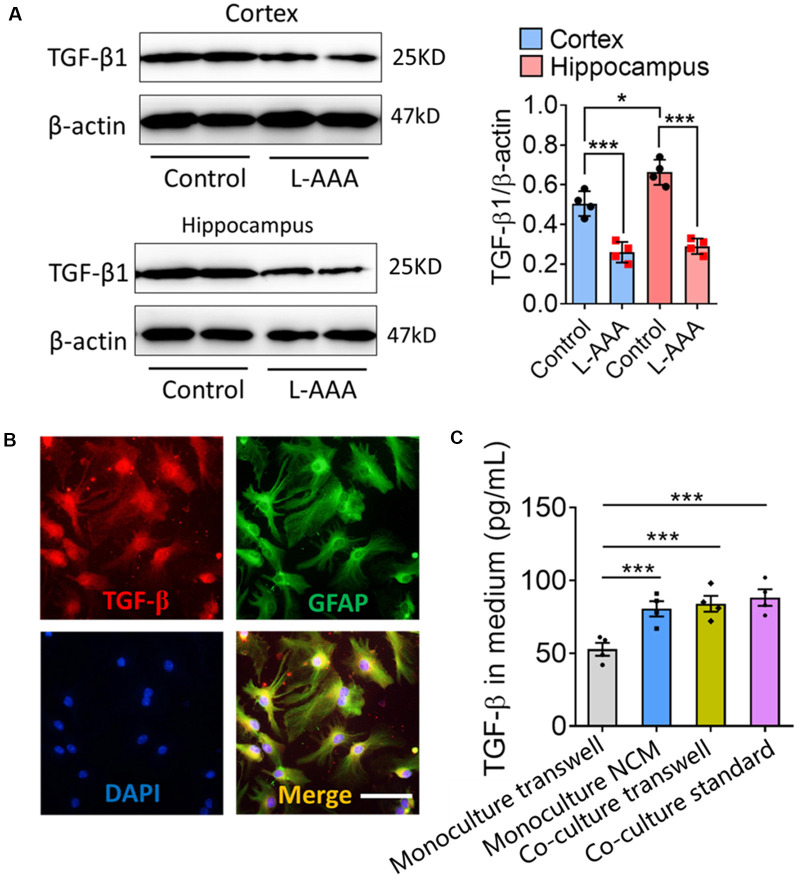
Astrocytes regulate the expression and secretion of TGF-β *in vivo* and *in vitro*. **(A)** Western blots showing TGF-β1 levels in the hippocampus and cortex from L-AAA-treated or control mice. TGF-β1 levels were normalized to those of β-actin. Data are mean ± SEM (*n* = 4/group). *P < 0.05 and ***P < 0.005 (two-way ANOVA with LSD test). **(B)** Immunofluorescence micrographs showing relative localization of TGF-β and GFAP^+^ cells. Scale bar, 10 μm. **(C)** Levels of TGF-β1 in the medium of microglia cultured alone in fresh medium or ACM, or cocultured with astrocytes in transwell dishes or standard dishes. Results of each group were obtained from four independent simples. Each sample was tested in triplicate and averaged. Data are presented as mean ± SEM, ***P < 0.005 (*n* = 4, one-way ANOVA with LSD).

Previous studies showed that TGF-β secreted by astrocytes contributes to microglial ramification (Schilling et al., 2001; Liu et al., 2011). Consistent with this, we found a higher level of TGF-β1 in the hippocampus than in the cortex, reflecting the different complexity of microglial branching (Figure 6A). Astrocyte ablation with L-AAA significantly reduced the expression of TGF-β1 in the hippocampus and cortex (Figure 6A), which we already showed to be associated with fewer and shorter branches of microglia (Figure 2).

Immunocytochemistry showed that TGF-β was released by GFAP^+^ cells (Figure 6B). The concentrations of TGF-β were significantly higher in the medium of microglia cultured alone in ACM or cocultured with astrocytes than in the medium of microglia cultured alone in fresh medium (Figure 6C).

**Figure 7:**
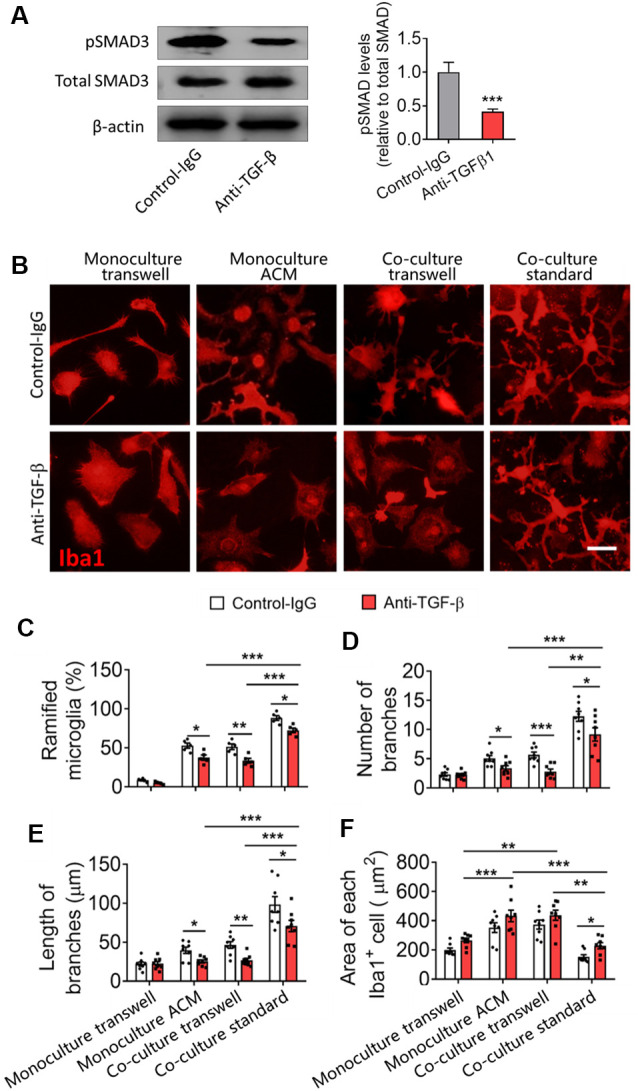
Astrocytes produce TGF-β that contributes to microglial ramification. **(A)** Western blotting showing changes in pSMAD3 in microglia cultured by ACM with or without anti-TGF-β antibody. **(B)** Fluorescence micrographs showing differences in microglial morphology with or without anti-TGF-β antibody. Microglia were labeled by Iba1 (red). Scale bar, 10 μm. **(C–F)** Percentage of ramified microglia (B), number **(C)** and length **(D)** of branches, and cell body area of microglia **(E)**. Results of each group were obtained from eight independent samples, and 4–5 micrographs were collected for each sample. All Iba1^+^ cells in each micrograph were measured. Each dot in the bar graph represents the average of each simple. Data are presented as mean ± SEM, *P < 0.05, **P < 0.01, and ***P < 0.005 (*n* = 8, unpaired two-tailed Student’s *t*-tests or two-way ANOVAs, followed by Tukey’s multiple-comparison test, where appropriate).

TGF-β neutralizing antibodies (anti-TGF-β) significantly decreased the pSMAD3 in primary microglia and decreased the percentage of ramified microglia, as well as the number and length of branches of microglial monocultures or cocultures (Figures 7A–F). Nevertheless, even in the presence of these antibodies, microglia cultured on astrocyte monolayers maintained a higher percentage of ramified microglia, more and longer branches, and smaller cell bodies than microglia cultured alone in ACM or cocultured with astrocytes in transwell dishes.

**Figure 8:**
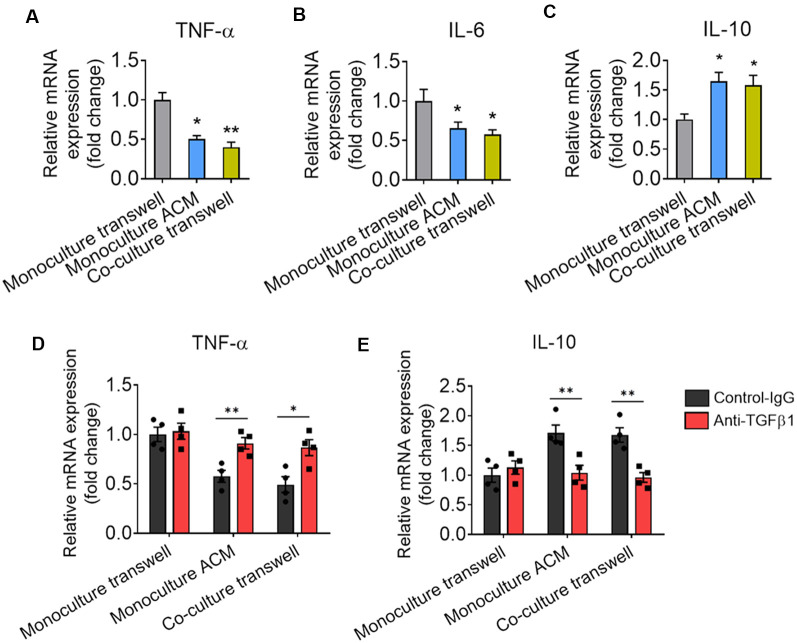
Astrocytes produce TGF-β that suppresses to microglia-mediated inflammatory response.** (A–C)** mRNA expression of TNF-α, IL-6, and IL-10 in microglia cultured alone, cocultured with astrocyte by transwell and cultured by ACM. Results of each group were obtained from four independent samples. Each sample is made in triplicate. (**D,E**) mRNA expression of TNF-α and IL-10 in microglia cultured alone, cocultured with astrocyte by transwell and cultured by ACM in the presence or absence of the anti-TGF-β antibody. Data are presented as mean ± SEM, *P < 0.05, **P < 0.01 (unpaired two-tailed Student’s *t*-tests, one-way or two-way ANOVAs, followed by Tukey’s multiple-comparison test, where appropriate).

We also found that astrocytes produce TGF-β that suppresses to microglia-mediated inflammatory response. Our data showed that the mRNA expression of TNF-α and IL-6 were decreased and the mRNA expression of IL-10 was increased in microglia cocultured with astrocyte by transwell or cultured by ACM when compared with microglia cultured alone (Figures 8A–C). TGF-β-neutralizing antibodies (anti-TGF-β) significantly increased the expression of TNF-α and decreased the expression of IL-10 in microglia (Figures 8D,E).

### Astrocytes Refine Microglial Ramification by Forming Glial Fibers That Create Traction

**Figure 9:**
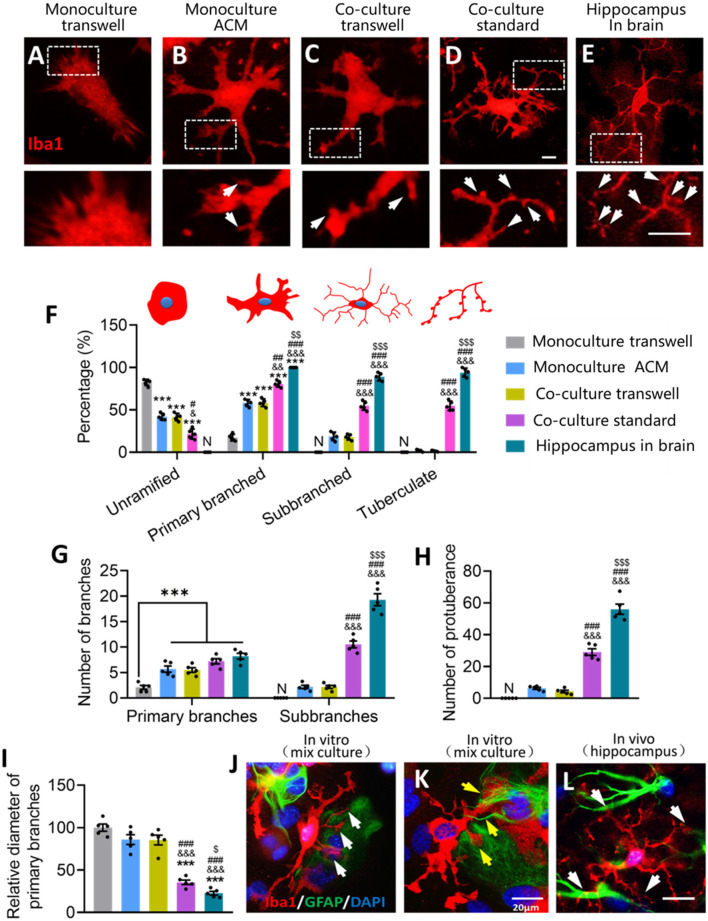
Glial fibers from astrocytes promote the formation of microglial sub-branches and protuberance. **(A–E)** Fluorescence micrographs showing differences in the branch microstructure of microglia under different conditions. Microglia were labeled by Iba1 (red). White arrows indicate protuberances of microglia. Scale bar, 5 μm. **(F)** Percentages of unramified microglia, primary branched microglia, sub-branched microglia, and tuberculated microglia under different conditions. **(G)** Numbers of primary branches and sub-branches in ramified microglia under different conditions. **(H)** Numbers of protuberance of microglia under different conditions. **(I)** Relative diameter of microglial primary branches under different conditions to the monoculture in fresh medium. **(J)** Fluorescence micrograph showing that microglia had more branches on the side where GFAP^+^ cells were present than the side where GFAP^+^ cells were absent. Microglia were labeled by Iba1 (red), astrocytes by GFAP (green), and nuclei by DAPI (blue). White arrows indicate the microglial ramification on the side where GFAP^+^ cells were present. Scale bar, 20 μm. **(K)** Fluorescence micrograph showing that glial fibers from astrocytes promote the formation of microglial sub-branches and protuberance. Yellow arrows point to glial fibers that wind and induce microglia branching. Scale bar, 20 μm. **(L)** Representative immunofluorescence micrograph showing distribution of the astrocyte marker GFAP and the microglial marker Iba1 in the hippocampus. White arrows indicate microglial branches growing toward GFAP^+^ cells. Scale bar, 20 μm. Results of each group were obtained from five independent samples, and 4–5 micrographs were collected for each sample. All Iba1^+^ cells in each micrograph were measured. Each dot in the bar graph represents the average of each simple. Data are presented as mean ± SEM, ***P < 0.005 vs. Monoculture transwell group, ^&^*P* < 0.05, ^&&^*P* < 0.01, and ^&&&^*P* < 0.005 vs. Co-culture transwell group, ^#^*P* < 0.05, ^##^*P* < 0.01, and ^###^*P* < 0.005 vs. monoculture ACM group, ^$^*P* < 0.05, ^$$^*P* < 0.01, and ^$$$^*P* < 0.005 vs. co-culture standard group [*n* = 5, N: no (the corresponding number is "0") unpaired two-tailed Student’s *t*-tests or two-way ANOVAs, followed by Tukey’s multiple-comparison test, where appropriate].

We then quantified the microstructure of microglial ramification. Primary microglia cultured alone had some filamentopodia on their surfaces, but no sub-branches or protuberances (Figure 9A). Microglia cultured alone in ACM or cocultured with astrocytes in transwell dishes had several primary branches and few protuberances (Figures 9B,C). Microglia cultured on astrocyte monolayers had refined ramifications, including primary branches, sub-branches, and protuberances that were similar to hippocampal microglia (Figures 9D,E).

Microglia cultured alone in ACM or cocultured with astrocytes showed a lower percentage of microglia that were unramified and a higher percentage with primary branches (Figure 9F). Microglia cultured on astrocyte monolayers showed even higher percentages of microglia with primary branches, sub-branches, and tuberculation, although these percentages were still lower than in hippocampal microglia (Figure 9F). Similar trends were seen in the numbers of primary branches and protuberances across the groups (Figures 9G,H). Relative to microglial monocultures in fresh medium, cocultures of microglia and astrocytes in standard dishes showed significantly smaller diameters of primary branches of microglia, but this was not seen in the case of microglia cultured alone in ACM or cocultured with astrocytes in transwell dishes (Figure 9I). These results suggest that astrocytes refine microglial ramification through contact-dependent mechanisms.

We explored this possibility further by examining the relationship between microglial ramification and GFAP. Microglia had more branches on the side where GFAP^+^ cells were present than on the side where GFAP^+^ cells were absent (Figure 9J). Astrocytes generated glial fibers to wrap around the branches of microglia (Figure 9K), and this phenomenon also occurred in brain microglia (Figure 9L). These results suggest that glial fibers from astrocytes promote the formation of sub-branches and protuberances in microglia.

## Discussion

Microglial dysfunction is associated with many neurodegenerative and psychological disorders (Mosher and Wyss-Coray, 2014). Microglial morphology is closely related to its function (Napoli and Neumann, 2009; Nayak et al., 2014), and the biological mechanisms that regulate microglial ramification are not well understood. In this study, we provide evidence that astrocytes regulate microglial ramification through a contact-independent pathway involving TGF-β, as well as a contact-dependent pathway involving glial fibers. Astrocyte-derived TGF-β promotes the formation of primary branches of microglia, while glial fibers promote the formation of microglial sub-branches and protuberances.

The morphology of microglia is remarkably plastic (Zhang et al., 2018). Microglia constantly adjust their shape and branches to adapt to the environment (Kettenmann et al., 2011). Capturing these environmental effects is difficult *in vitro*, as reflected in the well-known morphological differences between microglia in the brain or in primary cultures. Astrocytes, the most abundant cells type in the brain, play supportive and protective functions for neurons, and they regulate microglial activation and ramification (Norden et al., 2014). In this study, we found that Iba1^+^ cells had more and longer branches in the hippocampus, where GFAP^+^ cells were more abundant, than in the cortex. These data suggest that GFAP^+^ cells are involved in microglial ramification in the brain. Moreover, morphological differences of microglia in different brain regions may be related to the density of GFAP^+^ cells.

Processes of astrocytes extend in all directions and establish contacts with each other *via* gap junctions, thereby forming networks with other cells (Nedergaard et al., 2003). Our results showed that astrocyte ablation reduced the number and length of branches of Iba1^+^ cells and induced ameboid microglia in both mouse hippocampus and cortex. A possible reason for these results is that astrocyte ablation leads to the destruction of the physical structure that sustains microglial branching. Indeed, ablation of astrocytes can trigger depression-like psychological disorders that are associated with morphological changes of microglia in mice (Lee et al., 2013). Our data showed that ablation of astrocytes results in depressive-like behaviors, cognitive impairs, and neuroinflammation. These results suggest that astrocytes are involved in the maintenance of microglial multibranched morphology and neuropathology. However, further study is needed to rule out that our findings are due to microglial activation by L-AAA or damage to astrocytes.

In the *in vitro* experiment, we found that microglia cultured alone were mostly ameboid, but microglia cultured alone in ACM or together with astrocytes in transwell dishes developed several primary branches. The percentage of ramified microglia and the number and length of branches were significantly higher in microglia cocultured with astrocytes or cultured alone in ACM than in microglia cultured alone in fresh medium. Microglia cultured on astrocyte monolayers formed many delicate branches. Quantitative analysis showed that microglia cultured on astrocyte monolayers showed the highest percentage of ramified microglia, the most and longest branches, and the smallest cell bodies. These results suggested that astrocytes promote microglial ramification (Jha et al., 2019).

**Figure 10:**
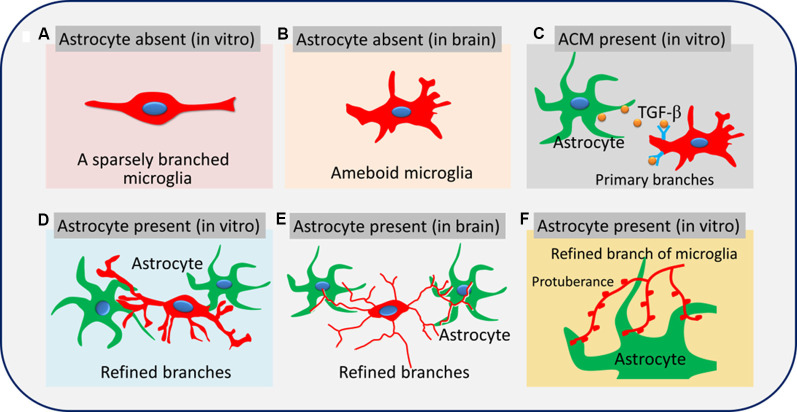
Astrocytes promote microglial ramification by releasing TGF-β and glial fibers. Microglia are sparsely branched in brain tissue or *in vitro* when astrocytes are absent **(A,B)**. Soluble factors secreted by astrocytes, such as TGF-β, promote the formation of primary branches of microglia **(C)**. Glial fibers from astrocytes promote the formation of refined branches (main branches are small in diameter with sub-branches and protuberances; **D–F**).

We also examined whether neurons also contributed to the remodeling of microglial branches and found no significant differences in the percentage of ramified microglia, number of branches, or soma area between microglia cultured alone in fresh or neuron-conditioned medium or microglia cocultured with neurons in transwell dishes. Our results suggest that astrocytes but not neurons regulate microglial branching through contact-dependent and -independent pathways. Astrocytes can promote microglia to form more branches through the contact-dependent pathway than contact-independent pathways (Figure 10). In addition to providing soluble media to regulate microglia branching, it may be more important to provide microglia with attachment and rivet structure.

Previous studies showed that TGF-β secreted by astrocytes contributes to microglial ramification (Schilling et al., 2001; Liu et al., 2011). In this study, we found a higher level of TGF-β in the hippocampus than in the cortex, reflecting the different complexity of microglial branching. Astrocyte ablation with L-AAA significantly reduced the expression of TGF-β in the hippocampus and cortex, which we already showed to be associated with fewer and shorter branches of microglia. The concentrations of TGF-β were significantly higher in the medium of microglia cultured alone in ACM or cocultured with astrocytes than in the medium of microglia cultured alone in fresh medium. Immunocytochemistry showed that TGF-β was released by GFAP^+^ cells. TGF-β-neutralizing antibodies significantly decreased the percentage of ramified microglia, as well as the number and length of branches of microglial monocultures or cocultures. These data suggested that astrocytes promote microglial ramification *via* contact-independent pathways by secreting TGF-β (Norden et al., 2014). It is has been reported that TGF-β1 promotes the neuroprotective functions of astrocytes against oxaliplatin neurotoxicity (Di Cesare Mannelli et al., 2015). It is noteworthy that under the condition of TGF-β neutralization, even if the number of branches of microglia cultured alone in ACM is reduced, the promoting effect of ACM on microglial ramification is not completely blocked. In addition to TGF-β, there are other factors that regulate microglial ramification. Our results showed that TGF-β also suppressed microglia-mediated inflammatory response. Study has shown that astrocytes secrete ATP, M-CSF, and GM-CSF to induce microglial ramification (Schilling et al., 2001).

Nevertheless, even in the presence of these antibodies, microglia cultured on astrocyte monolayers maintained a higher percentage of ramified microglia, more and longer branches, and smaller cell bodies than microglia cultured alone in ACM or cocultured with astrocytes in transwell dishes. It is crucial for astrocytes to regulate microglial branching through contact-dependent pathways. At the same time, we found that culturing microglia on astrocyte monolayers induced delicate branching *in vitro*, highlighting the role of physical attachment between microglia and astrocytes in regulating microglial branching. The length and number of branches determine the sensitivity and range of microglia to monitor the environment in the brain. We found that astrocytes generate glial fibers that wrap around the branches of microglia. These glial fibers may help microglia extend their branches by providing traction, and it may improve the transmission of information between cells.

## Conclusions

Astrocytes promote microglial ramification *via* TGF-β and glial fibers. These findings provide new insights into the interaction between microglia and astrocytes. They also suggest that modulating astrocytes may be a therapeutic strategy against neurological diseases involving microglia dysfunction.

## Data Availability Statement

The raw data supporting the conclusions of this article will be made available by the authors, without undue reservation.

## Ethics Statement

The animal study was reviewed and approved by Guizhou University of Traditional Chinese Medicine.

## Author Contributions

JZ, LZ, and TZ conceived and designed the study and wrote the manuscript. XJ and YZ performed the primary microglial culture and testing for phenotypic markers. YQ and SY performed the neural stem/precursor cell analysis, neuronal culture, immunofluorescence studies, and cytokine assays. CX performed statistical analysis. All authors approved the final version of the manuscript.

## Conflict of Interest

The authors declare that the research was conducted in the absence of any commercial or financial relationships that could be construed as a potential conflict of interest.

## Abbreviations

CNS: central nervous system
GFAP: glial fibrillary acidic protein
L-AAA: L-alpha-aminoadipic acid
TGF-β: transforming growth factor-β
ACM: astrocyte-conditioned medium.
