# Supplementary material for: Mouse Astrocytes Promote Microglial Ramification by Releasing TGF-β and Forming Glial Fibers
Source: Front Cell Neurosci. 2020 Jul 10;14:195. doi: 10.3389/fncel.2020.00195 (PMC7366495; doi:10.3389/fncel.2020.00195)
Supplement: Supplementary file 1 [file Data_Sheet_1.PDF]

Supplementary Figures and Legends

Figure S1. Morphological differences between microglia *in vivo* and *in vitro*.

A: Representative micrographs showing hippocampal microglia and primary microglia stained with Iba1 (red) and DAPI (blue). Scale bar, 10 μm.

B–D: Cell body area of Iba1$^+$ cells (B) as well as number (C) and length (D) of branches of microglia *in vivo* and *in vitro*.

Results were obtained from 4 independent samples, and 5 micrographs were collected from each sample. All Iba1$^+$ cells in each micrograph were measured. Each dot in the bar graph represents the average of all Iba1$^+$ cells in a micrograph. Data are presented as mean ± SEM. ***P < 0.005 vs. in vivo (unpaired *t* test).

Figure S2. A supplement to figure 2.

A and **B**: Quantification of the area of GFAP$^+$ cells in the hippocampus and cortex.

C: Quantification of the number of Iba1$^+$ cells in the hippocampus and cortex. Data are presented as mean ± SEM. ***P < 0.005 (unpaired *t* test).

Figure S3. Neurons do not significantly affect ramification of microglia in culture.

A: Schematic diagrams showing four methods to culture primary microglia: cultured alone, con-cultured with neuron by transwell, cultured by neuron conditioned medium (NCM) and mix cultured with neuron.

B: Fluorescence micrographs showing different microglial morphology under

different conditions. Microglia were labeled by Iba1 (red), neurons by MAP2 (green), and nuclei by DAPI (blue).

C–E: Percentage of ramified microglia (C), number of branches (D) and cell body area of microglia (E). Results of each group were obtained from 5 independent samples, and 5-6 micrographs were collected for each sample. All $Iba1^+$ cells in each micrograph were measured. Each dot in the bar graph represents the average of each sample. Data are presented as the mean ± SEM (n=5, one-way ANOVA with LSD).
