# Supplementary figures and images for: Mouse Astrocytes Promote Microglial Ramification by Releasing TGF-β and Forming Glial Fibers
Source: Front Cell Neurosci. 2020 Jul 10;14:195. doi: 10.3389/fncel.2020.00195 (PMC7366495; doi:10.3389/fncel.2020.00195)

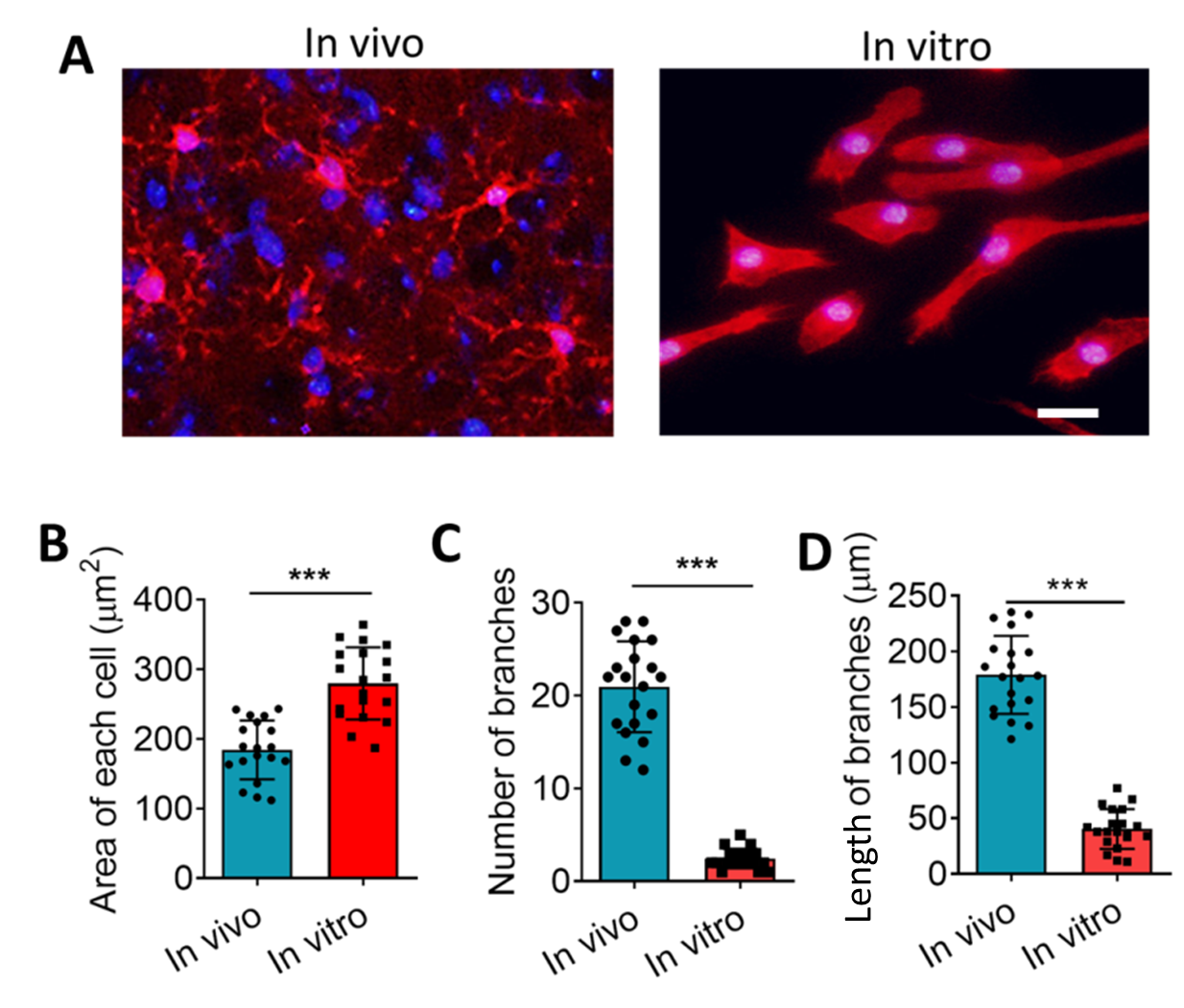

Supplement: Supplementary file 2 [file Image_1.TIF]

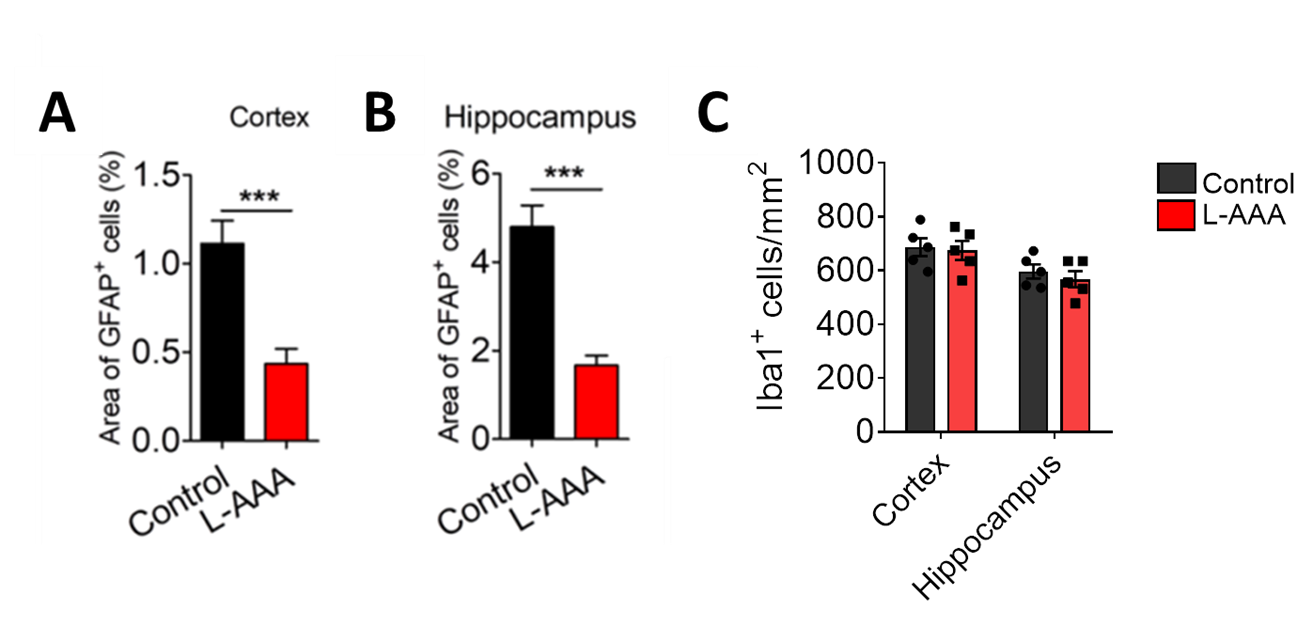

Supplement: Supplementary file 3 [file Image_2.TIF]

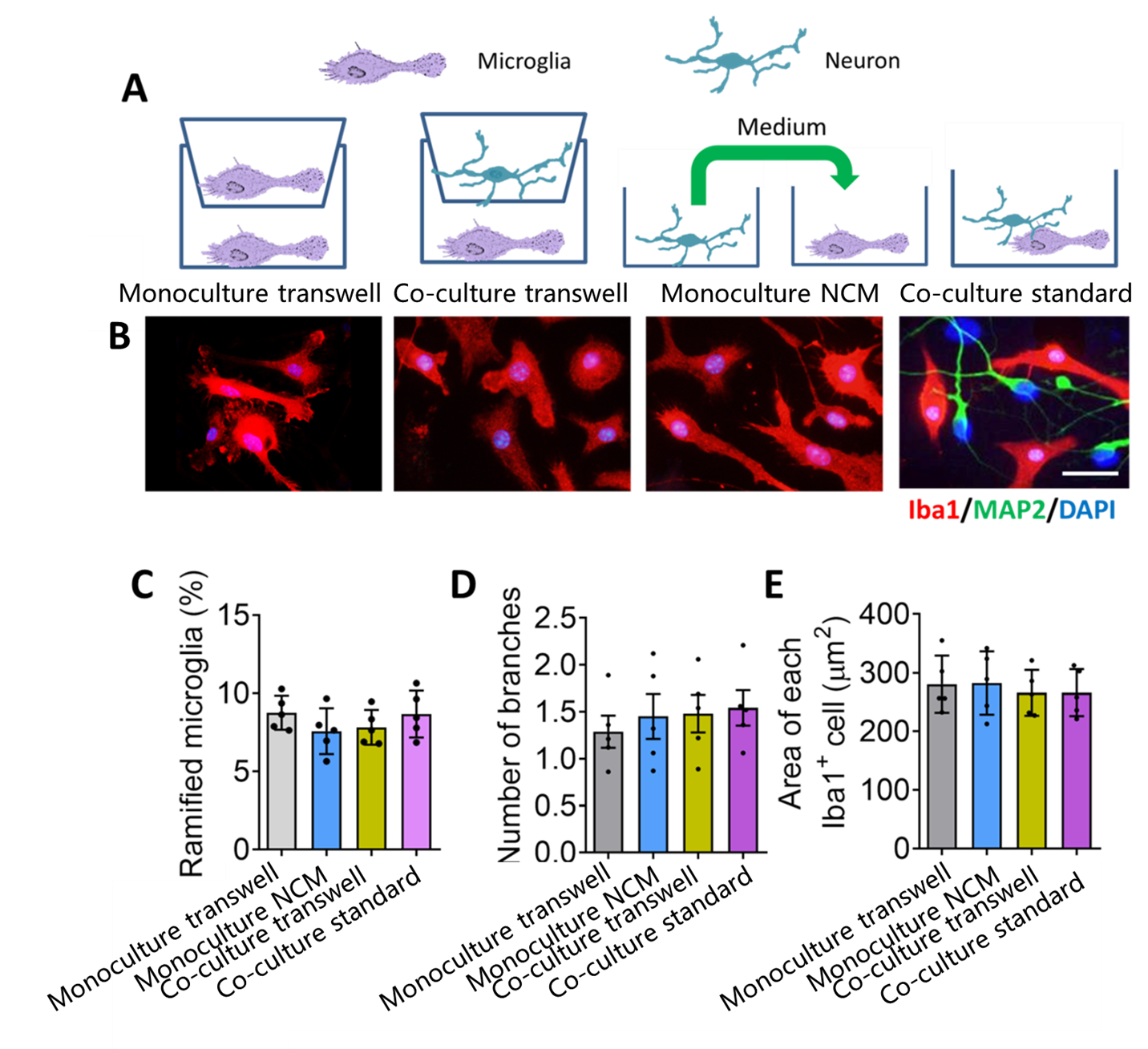

Supplement: Supplementary file 4 [file Image_3.TIF]
